# Long-term effects of medical management on growth and weight in individuals with urea cycle disorders

**DOI:** 10.1038/s41598-020-67496-3

**Published:** 2020-07-20

**Authors:** Roland Posset, Sven F. Garbade, Florian Gleich, Andrea L. Gropman, Pascale de Lonlay, Georg F. Hoffmann, Angeles Garcia-Cazorla, Sandesh C. S. Nagamani, Matthias R. Baumgartner, Andreas Schulze, Dries Dobbelaere, Marc Yudkoff, Stefan Kölker, Matthias Zielonka, Urea Cycle Disorders Consortium (UCDC), Nicholas Ah Mew, Susan A. Berry, Shawn E. McCandless, Curtis Coughlin, Gregory Enns, Renata C. Gallagher, Lindsay C. Burrage, Jennifer Seminara, Cary O. Harding, Peter Burgard, Cynthia Le Mons, J. Lawrence Merritt, Tamar Stricker, Jirair K. Bedoyan, Gerard T. Berry, George A. Diaz, Derek Wong, Mendel Tuchman, Susan Waisbren, James D. Weisfeld-Adams, European registry and network for Intoxication type Metabolic Diseases (E-IMD), Alberto B. Burlina, Elisa Leão Teles, Consuelo Pedrón-Giner, Allan M. Lund, Carlo Dionisi-Vici, Monique Williams, Ulrike Mütze, Daniela Karall, Javier Blasco-Alonso, Maria L. Couce, Jolanta Sykut-Cegielska, Persephone Augoustides-Savvopoulou, Angeles Ruiz Gomez, Ivo Barić, Manuel Schiff, Yin-Hsiu Chien, Martin Lindner, Brigitte Chabrol, Anastasia Skouma, Jiri Zeman, Etienne Sokal, René Santer, Francois Eyskens, Peter Freisinger, Luis Peña-Quintana, Dominique Roland, Elisenda Cortès-Saladelafont, Maja Djordjevic

**Affiliations:** 1 Center for Pediatric and Adolescent Medicine, Division of Pediatric Neurology and Metabolic Medicine, University Hospital Heidelberg, Im Neuenheimer Feld 430, 69120 Heidelberg, Germany; 2 Children’s National Health System, Washington, DC USA; 3 Hôpital Necker-Enfants Malades, Assistance Publique-Hôpitaux de Paris, Service de Maladies Metaboliques (MaMEA), filière G2M, Université Paris-Descartes, Paris, France; 4 Hospital San Joan de Deu, Institut Pediàtric de Recerca. Servicio de Neurologia and CIBERER, ISCIII, Barcelona, Spain; 5 Department of Molecular and Human Genetics, Baylor College of Medicine and Texas Children’s Hospital, Houston, TX USA; 6 University Children’s Hospital Zurich and Children’s Research Center, Zurich, Switzerland; 7 University of Toronto and the Hospital for Sick Children, Toronto, ON Canada; 8 Centre de Référence Maladies Héréditaires du Métabolisme de L’Enfant Et de L’Adulte, Jeanne de Flandre Hospital, CHRU Lille, and Faculty of Medicine, University Lille 2, Lille, France; 9 School of Medicine and Children’s Hospital of Philadelphia, University of Pennsylvania, Philadelphia, PA USA; 10 Heidelberg Research Center for Molecular Medicine (HRCMM), Heidelberg, Germany; 11 University of Minnesota, Minneapolis, MN USA; 12 Children’s Hospital Colorado and University of Colorado School of Medicine, Aurora, CO USA; 13 Stanford Children’s Health, Palo Alto, CA USA; 14 University of California, San Francisco, CA USA; 15 Oregon Health and Science University, Portland, OR USA; 16 National Urea Cycle Disorders Foundation (NUCDF), Pasadena, USA; 17 University of Washington and Seattle Children’s Hospital, Seattle, WA USA; 18 Center for Human Genetics and Department of Genetics and Genome Sciences, University Hospitals Cleveland Medical Center and Case Western Reserve University, Cleveland, OH USA; 19 Harvard Medical School and Boston Children’s Hospital, Boston, MA USA; 20 Mount Sinai School of Medicine, Department of Genetics and Genomics Sciences, New York City, NY USA; 21 David Geffen School of Medicine at UCLA, Los Angeles, CA USA; 22 Division of Inherited Metabolic Diseases, Reference Centre Expanded Newborn Screening, Department of Woman’s and Child’s Health, University Hospital Padova, Padua, Italy; 23 Unidade de Doenças Metabólicas, Serviço de Pediatria, Hospital de S. João, EPE, Porto, Portugal; 24 Division of Gastroenterology and Nutrition, Hospital Infantil Universitario Niño Jesús, Madrid, Spain; 25 Centre for Inherited Metabolic Diseases, Departments of Paediatrics and Clinical Genetics, Copenhagen University Hospital, Copenhagen, Denmark; 26 Ospedale Pediatrico Bambino Gesù, U.O.C. Patologia Metabolica, Rome, Italy; 27 Erasmus MC-Sophia Kinderziekenhuis, Erasmus Universiteit Rotterdam, Rotterdam, The Netherlands; 28 Clinic for Pediatrics, Division of Inherited Metabolic Disorders, Medical University of Innsbruck, Innsbruck, Austria; 29 Hospital Materno-Infantil, Málaga, Spain; 30 Hospital Clinico Universitario de Santiago de Compostela, Metabolic Unit, Department of Pediatrics, Santiago de Compostela, Spain; 31 Department of Inborn Errors of Metabolism and Paediatrics, Institute of Mother and Child, Warsaw, Poland; 32 1st Pediatric Department, Aristotle University of Thessaloniki, Thessaloniki, Greece; 33 Metabolic Diseases Unit, Pediatric Neurology Department, Hospital Universitario Son Dureta, Palma de Mallorca, Spain; 34 University Hospital Center Zagreb and University of Zagreb, School of Medicine, Zagreb, Croatia; 35 Robert-Debré University Hospital, Reference Center for Inborn Errors of Metabolism, Paris, France; 36 Department of Medical Genetics and Pediatrics, National Taiwan University Hospital, National Taiwan University College of Medicine, Taipei, Taiwan; 37 University Children’s Hospital Frankfurt, Frankfurt, Germany; 38 Centre de Référence des Maladies Héréditaires du Métabolisme, Service de Neurologie, Hôpital d’Enfants, CHU Timone, Marseille, France; 39 Institute of Child Health, Athens, Greece; 40 Department of Paediatrics, First Faculty of Medicine and General Faculty Hospital, Prague, Czech Republic; 41 Cliniques Universitaires St Luc, Université Catholique de Louvain, Service Gastroentérologie and Hépatologie Pédiatrique, Brussels, Belgium; 42 University Medical Center Eppendorf, Hamburg, Germany; 43 Universitair Ziekenhuis Antwerpen, Antwerp, Belgium; 44 Klinik für Kinder- und Jugendmedizin, Klinikum am Steinenberg, Reutlingen, Germany; 45 Gastroenterology and Nutrition Unit Complejo Hospitalario Universitario Insular-Materno Infantil, CIBEROBN, Las Palmas de Gran Canaria University, Las Palmas, Spain; 46 Institut de Pathologie et de Génétique ASBL, Centre Agréé des Maladies Héréditaires du Métabolisme, Centre de Génétique Humaine, Gosselies, Belgium; 47 Department of Paediatrics, Universitat Autònoma de Barcelona, Badalona, Spain; 48 Institut za zdravstvenu zastitu majke i deteta Srbije “Dr Vukan Cupic”, Radoja Dakica Street 6-8, and University of Belgrade, School of Medicine, Novi Beograd, Republic of Serbia

**Keywords:** Genetics, Diseases, Medical research, Neurology, Risk factors, Signs and symptoms

## Abstract

Low protein diet and sodium or glycerol phenylbutyrate, two pillars of recommended long-term therapy of individuals with urea cycle disorders (UCDs), involve the risk of iatrogenic growth failure. Limited evidence-based studies hamper our knowledge on the long-term effects of the proposed medical management in individuals with UCDs. We studied the impact of medical management on growth and weight development in 307 individuals longitudinally followed by the Urea Cycle Disorders Consortium (UCDC) and the European registry and network for Intoxication type Metabolic Diseases (E-IMD). Intrauterine growth of all investigated UCDs and postnatal linear growth of asymptomatic individuals remained unaffected. Symptomatic individuals were at risk of progressive growth retardation independent from the underlying disease and the degree of natural protein restriction. Growth impairment was determined by disease severity and associated with reduced or borderline plasma branched-chain amino acid (BCAA) concentrations. Liver transplantation appeared to have a beneficial effect on growth. Weight development remained unaffected both in asymptomatic and symptomatic individuals. Progressive growth impairment depends on disease severity and plasma BCAA concentrations, but cannot be predicted by the amount of natural protein intake alone. Future clinical trials are necessary to evaluate whether supplementation with BCAAs might improve growth in UCDs.

## Introduction

Urea cycle disorders (UCDs) are rare inherited metabolic diseases, consisting of 5 enzymopathies, 2 transporters and 2 associated cofactor-producing enzymes, with an estimated overall prevalence of 1 in 35,000 to 52,000 newborns^1^. The phenotypic spectrum is wide ranging from severe life-threatening hyperammonemic decompensations within the first 28 days of life (EO, early onset) to mild or moderate chronic hyperammonemic conditions reflected by a heterogeneous clinical spectrum such as lethargy, headache, hepatological, gastrointenstinal and neurological or psychiatric symptoms any time after the neonatal period (LO, late onset)^2–4^. Long-term dietary management is challenging and consists of a low protein diet with or without supplementation of essential amino acids (EAAs), vitamins, trace elements and/or single amino acids, while pharmacological long-term treatment with nitrogen scavengers, i.e. sodium benzoate (BZA) and sodium or glycerol phenylbutyrate (PBA), aims at improving the urinary excretion of waste nitrogen via alternative pathways to reduce the frequency and severity of hyperammonemic episodes and hence improve survival and clinical outcomes of affected individuals^2, 5^. However, a protein restricted diet, defined by a natural protein intake below 100% of the World Health Organization (WHO) safe values recommendations^6^, as well as depletion of branched-chain amino acids (BCAAs), the latter aggravated by the administration of PBA^7^, are thought to impair growth and weight development. Recently, survey-based studies from the UK and Europe found that dietary treatment practices vary widely between different countries, particularly with regard to daily protein prescriptions in early childhood, use of EAAs and BCAA supplementation, and nutritional support with vitamins, trace elements, minerals and essential fatty acids. Moreover, clinical trials investigating dietary treatment outcomes between different severity-adjusted UCD subgroups with regard to clinical endpoints, such as growth or metabolic stability, are still lacking^8–10^. Thus, current recommendations for dietary and pharmacological long-term management remain inconclusive due to missing longitudinal studies evaluating the (adverse) effects of current treatment principles^2,5,8,9^.

Based on a combined and comparative data analysis approach between large international multicenter registry studies from North America [Urea Cycle Disorders Consortium (UCDC; https://www.rarediseasesnetwork.org/cms/ucdc)] and Europe [European registry and network for Intoxication type Metabolic Disease (E-IMD; https://www.eimd-registry.org/)], a new strategy for clinical outcome research in the field of rare diseases became lately available^11^. This enabled us to evaluate the impact of long-term management on the cognitive outcome of individuals with UCDs, providing further evidence for future recommendations^12^. Due to the low clinical evidence with regard to the long-term (adverse) effects of medical treatment, we studied longitudinal data from the UCDC and E-IMD databases to address this shortcoming after two decades of systematic data collection.

To this end, we investigated whether individuals with UCDs suffer from intrauterine or postnatal weight and growth retardation, and whether medical management—as currently performed in symptomatic individuals with UCDs in North America and Europe—is safe or leads to an impaired development with regard to anthropometrical endpoints.

## Results

### Anthropometrical parameters at birth are within normal range

**Figure 1:**
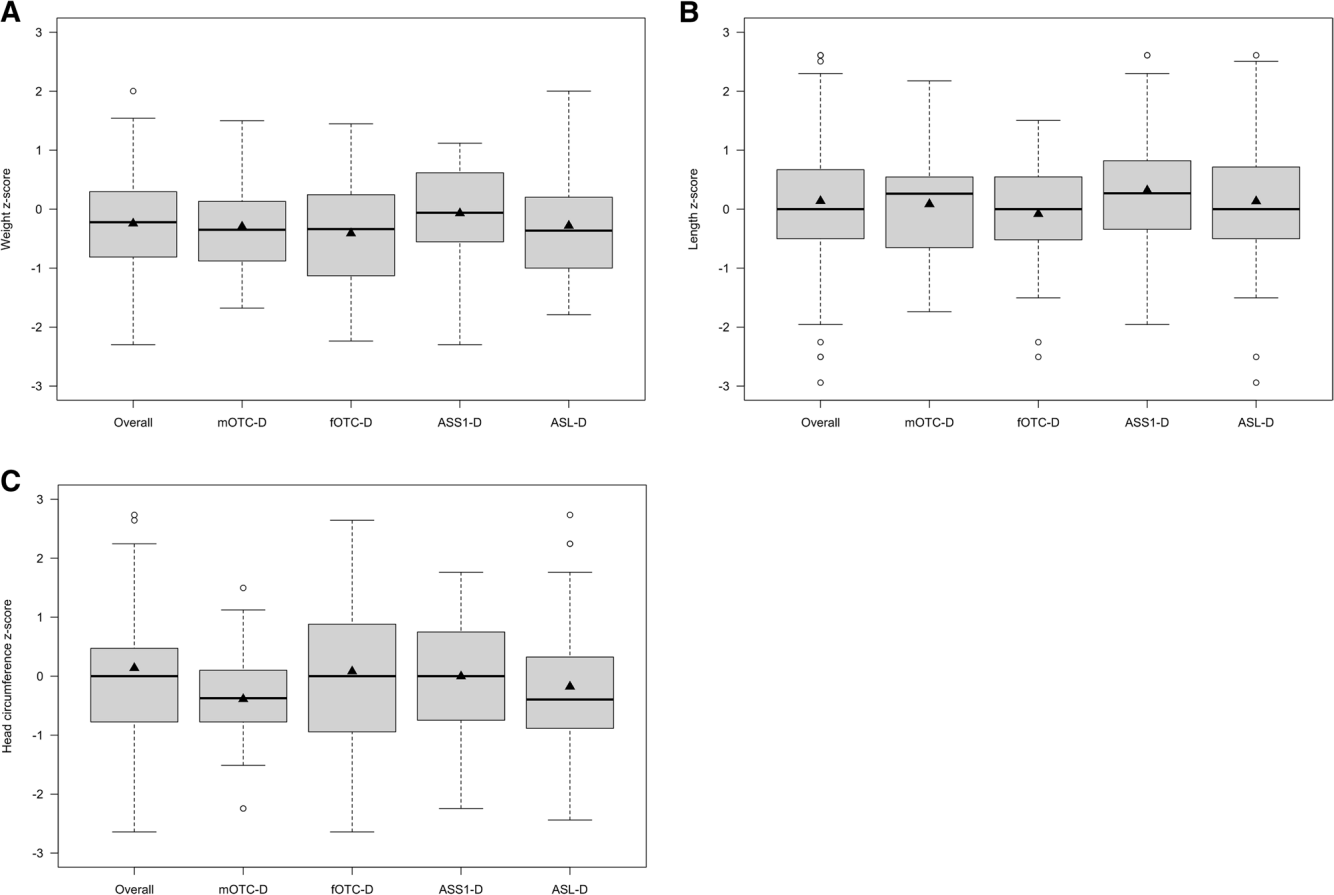
Intrauterine development of 205 individuals with UCDs is unaffected. Z-scores of weight (**A**), length (**B**), and head circumference (**C**) of 205 individuals with UCDs at birth. Mean z-scores for weight (z-score: − 0.24), length (z-score: 0.14) and head circumference (z-score: − 0.13) are overall within the normal range. Various diseases do not differ with regard to weight (*p* = 0.20; ANOVA), length (*p* = 0.26; ANOVA), and head circumference (*p* = 0.11; ANOVA). Data are shown as median (black line) and mean (triangle), length of the box corresponds to interquartile range (IQR), upper and lower whiskers correspond to max. 1.5 × IQR, each point represents an outlier. Descriptive characteristics are presented separately in Supplementary Table S1. ASL-D, argininosuccinate lyase deficiency; ASS1-D, argininosuccinate synthetase 1 deficiency; fOTC-D, female ornithine transcarbamylase deficiency; mOTC-D, male ornithine transcarbamylase deficiency.

Overall, 205 individuals with UCDs [male ornithine transcarbamylase deficiency (mOTC-D): n = 54, 26.4%; female OTC-D (fOTC-D): n = 39, 19.0%; argininosuccinate synthetase 1 deficiency (ASS1-D): n = 64, 31.2%; argininosuccinate lyase deficiency (ASL-D): n = 48, 23.4%] have a mean z-score within the normal range for birth weight, length and head circumference [mean z-score (birth weight): − 0.24; mean z-score (birth length): 0.14; mean z-score (birth head circumference): − 0.13] (Fig. 1A–C). Neither disease (mOTC-D, fOTC-D, ASS1-D, ASL-D) nor sex (male, female) showed a specific impact on birth weight (disease: *p* = 0.18, sex: *p* = 0.15; ANOVA), birth length (disease: *p* = 0.30, sex: *p* = 0.57; ANOVA), or birth head circumference (disease: *p* = 0.74, sex: *p* = 0.09; ANOVA).

### Asymptomatic individuals have a normal postnatal growth and weight development

**Figure 2:**
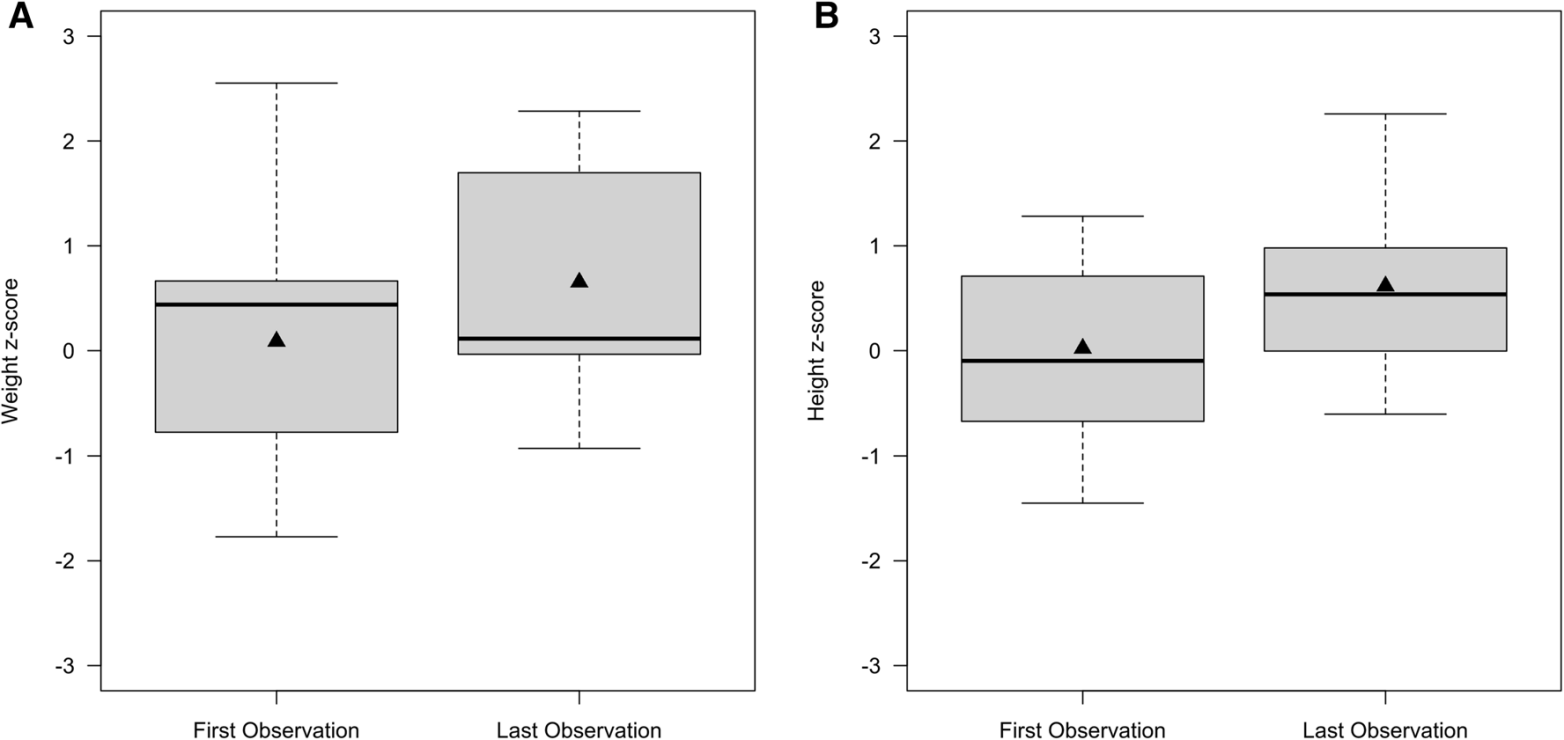
Postnatal weight development and linear growth of 11 asymptomatic UCDs is normal. Preschool children were observed during a period of approximately 3 years and both weight (**A**) and height (**B**) did not differ between the first [mean z-score (weight): 0.09; mean z-score (height): 0.02] and last [mean z-score (weight): 0.65; mean z-score (height): 0.62] observation (each *p* > 0.05; *t*-test). Moreover, weight and height were within the normal range. Data are shown as median (black line) and mean (triangle), length of the box corresponds to IQR, upper and lower whiskers correspond to max. 1.5 × IQR. Descriptive characteristics are presented separately in Supplementary Table S1.

Next, we studied the postnatal anthropometrical development of asymptomatic and untreated individuals with UCDs. Overall 11 individuals (mOTC-D: n = 2, 18.2%; fOTC-D: n = 3, 27.3%; ASS1-D: n = 4, 36.3%; ASL-D: n = 2, 18.2%) with a mean individual observation period of 2.95 years (min: 1.59 years; max: 4.54 years) were investigated. Mean age at first visit was 1.56 years (min: 0.01 years; max: 8.71 years) and mean age at last visit corresponded to 4.51 years (min: 1.96 years; max: 12.27 years), reflecting a preschool population of UCDs. Neither disease nor age showed an impact on postnatal weight development (disease: *p* = 0.33, age: *p* = 0.27; ANOVA) and linear growth (disease: *p* = 0.72, age: *p* = 0.16; ANOVA) within the observation period (Fig. 2A,B), highlighting that asymptomatic individuals with UCDs have a normal postnatal anthropometrical development.

### Symptomatic individuals have a risk of postnatal growth retardation

**Figure 3:**
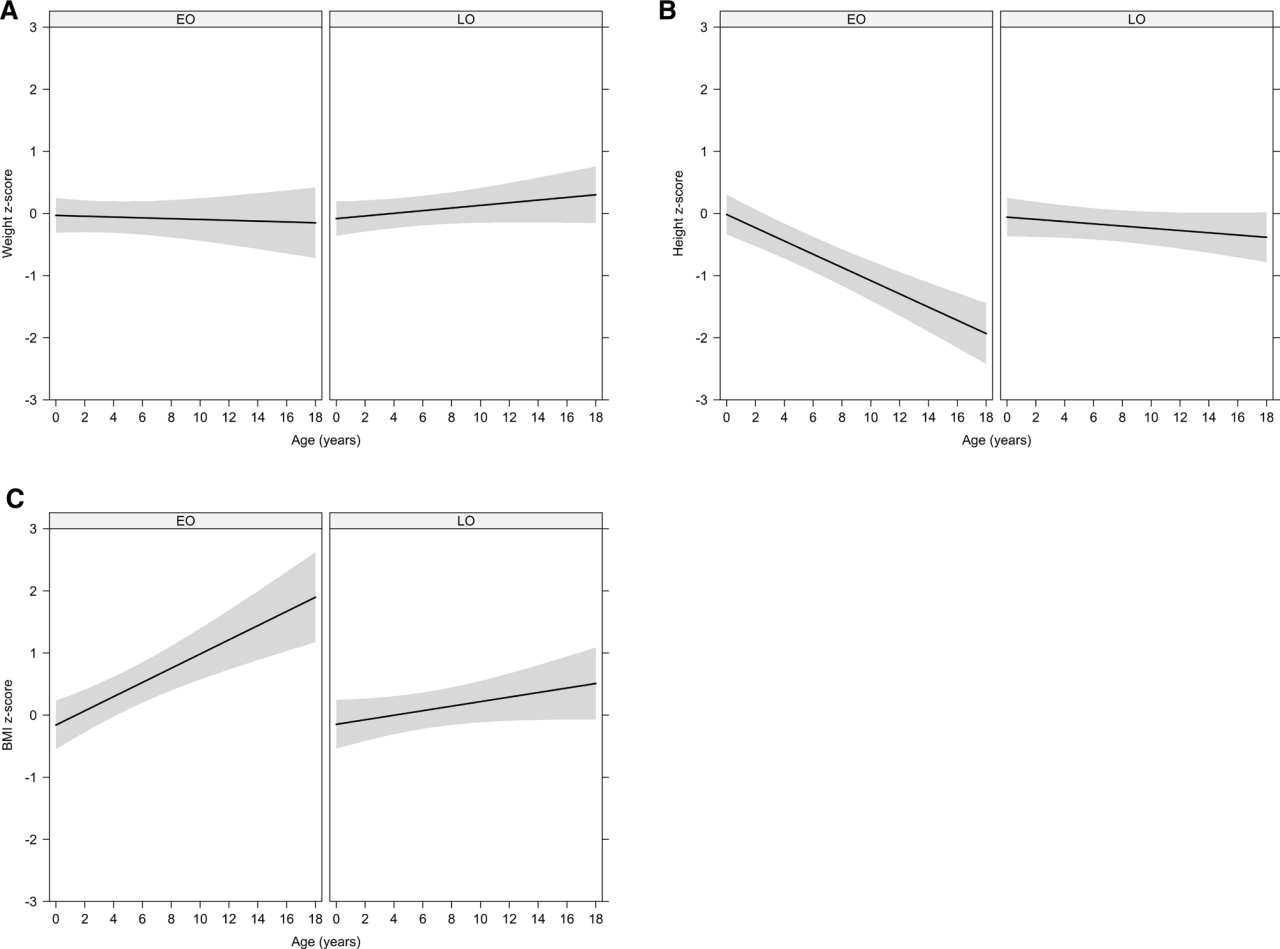
Symptomatic individuals (n = 130) have normal weight development but abnormal linear growth. Prepubertal children were observed during a period of approximately 5 years. Weight (**A**) was not affected neither by age (*p* = 0.41; LME ANOVA), nor by disease onset (*p* = 0.77; LME ANOVA) or specific diseases (*p* = 0.71; LME ANOVA). β-Coefficients did not differ between EO (β = − 0.01) and LO (β = 0.02; *p* = 0.23, LME) individuals. Height (**B**) however, was affected by age (*p* < 0.001; LME ANOVA), disease onset (*p* = 0.03; LME ANOVA) and interaction of both (*p* < 0.001; LME ANOVA), suggesting that disease severity—as reflected by EO individuals—is associated with impaired growth over time. In line, β-coefficients differed between EO (β = − 0.11) and LO (β = − 0.02; *p* < 0.001, LME) individuals. Accordingly, BMI (**C**) was determined by age (*p* < 0.001; LME ANOVA) and interaction between age and disease onset (*p* = 0.02; LME ANOVA), indicating that BMI increases with age for the EO but not the LO group. Gray lines are fitted weight, height, and BMI values from the LME model; the gray shaded area corresponds to 95% confidence interval. Descriptive characteristics are presented separately in Supplementary Table S1. BMI, body mass index; EO, early onset; LO, late onset.

To assess whether symptomatic individuals had a risk of postnatal weight and growth retardation, we evaluated 130 symptomatic individuals (mOTC-D: n = 33, 25.4%; fOTC-D: n = 42, 32.3%; ASS1-D: n = 25, 19.2%; ASL-D: n = 30, 23.1%) receiving conservative management during a mean individual observation period of 4.81 years (min: 1.00 years; max: 12.44 years). Overall, the observation period spanned a time frame within prepubertal childhood from mean age at first visit of 6.33 years (min: 0.03 years; max: 15.52 years) to mean age at last visit of 11.14 years (min: 1.27 years; max: 17.99 years). Since early disease onset was associated with higher disease severity^10^, we investigated whether specific diseases were associated with higher initial peak plasma ammonium concentrations (NH_4_^+^_max_) in our study population. Interaction between disease onset (EO vs. LO) and a specific disease (mOTC-D, fOTC-D, ASS1-D, ASL-D) was not significant (*p* = 0.86; LME ANOVA). Furthermore, neither disease onset (*p* = 0.77; LME ANOVA), nor a specific disease (*p* = 0.71; LME ANOVA) or age (*p* = 0.41; LME ANOVA) had a measurable impact on weight gain during the observation period, demonstrating that current conservative management does not impair weight development of symptomatic individuals with UCDs (Fig. 3A). In contrast, symptomatic individuals suffered from postnatal growth retardation. This was not associated with a specific UCD (*p* = 0.45; LME ANOVA), while disease onset (*p* = 0.03; LME ANOVA), age (*p* < 0.001; LME ANOVA), and interaction between disease onset and age (*p* < 0.001; LME ANOVA) had a significant impact on linear growth indicating that particularly EO patients of any UCD studied suffered from postnatal growth retardation (Fig. 3B). Accordingly, body mass index (BMI) of affected individuals increased over time for the EO but not LO group as indicated by a significant interaction between disease onset and age (*p* = 0.02; LME ANOVA; Fig. 3C).

### Postnatal growth retardation in symptomatic individuals with UCDs is not associated with a protein restricted diet

**Figure 4:**
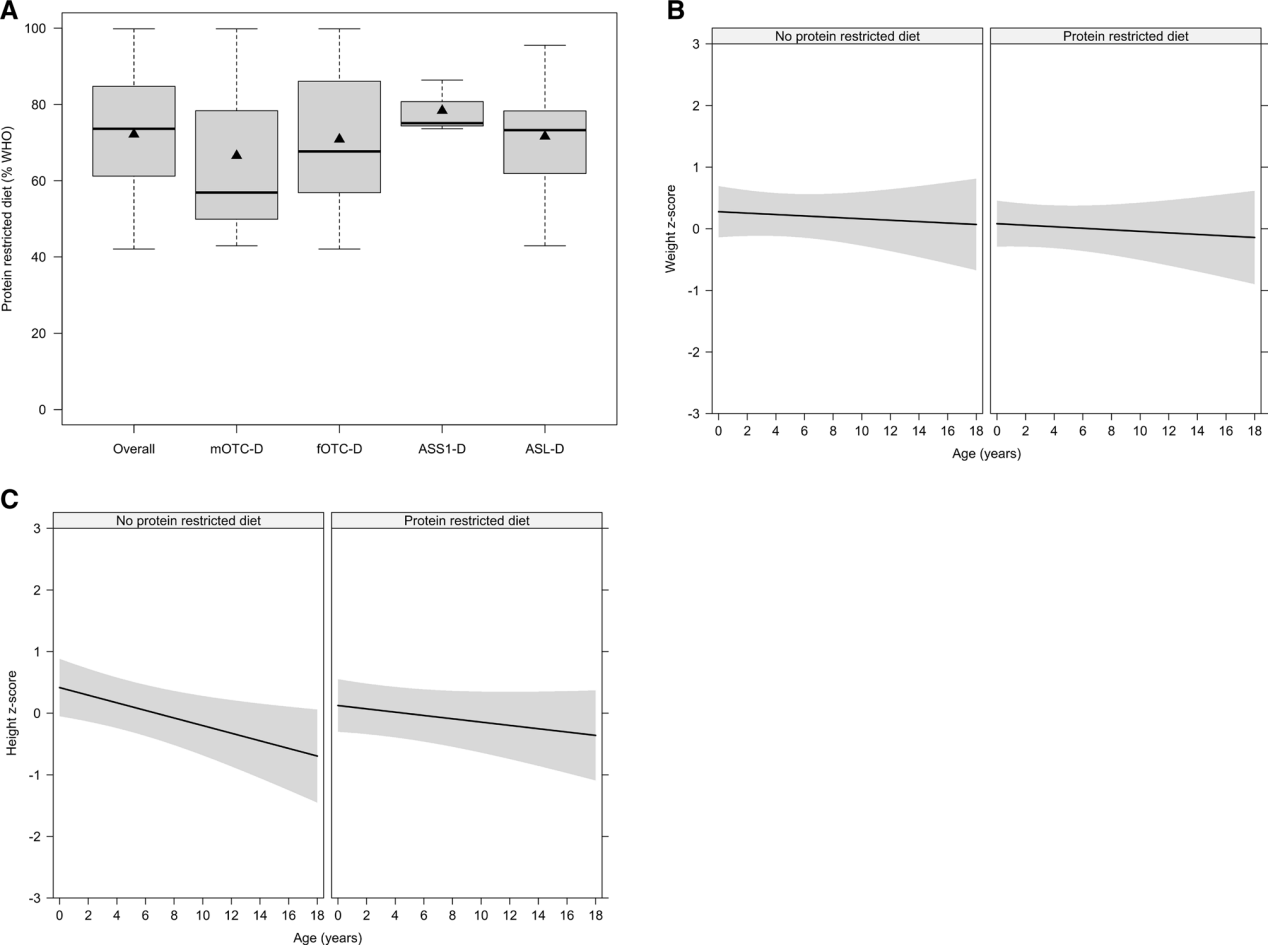
Impaired growth is not associated with the use of protein restricted diet as part of medical management in 46 symptomatic UCDs. Preschool children receiving a protein restricted diet were similarly restricted (*p* = 0.28; ANOVA) with regard to their mean natural protein intake. Data are shown as median (black line) and mean (triangle), length of the box corresponds to IQR, upper and lower whiskers correspond to max. 1.5 × IQR (**A**). During an observation period of approximately 3 years individuals with (n = 23) or without (n = 23) a protein restricted diet did not differ (each *p* > 0.05; LME ANOVA) with regard to their normal weight development (**B**) and impaired linear growth (**C**). β-coefficients did not differ between individuals with or without a protein restricted diet neither for weight development (protein restricted diet: β = − 0.01, no protein restricted diet: β = − 0.01; *p* = 0.98, LME; **B**) nor for linear growth (protein restricted diet: β = − 0.03; no protein restricted diet: β = − 0.06; *p* = 0.28; LME; **C**). Gray lines are fitted weight and height values from the LME model; the gray shaded area corresponds to 95% confidence interval (**B**, **C**). Descriptive characteristics are presented separately in Supplementary Table S1. ASL-D, argininosuccinate lyase deficiency; ASS1-D, argininosuccinate synthetase 1 deficiency; fOTC-D, female ornithine transcarbamylase deficiency; mOTC-D, male ornithine transcarbamylase deficiency.

To evaluate whether the observed postnatal growth retardation in symptomatic individuals was due to the protein restricted diet or the caloric intake as part of the conservative management, we studied 46 severity-adjusted UCD individuals (mOTC-D: n = 9, 19.6%; fOTC-D: n = 11, 23.9%; ASS1-D: n = 11, 23.9%; ASL-D: n = 15, 32.6%) with sufficient information on biochemical and therapy-related longitudinal data, comprising a mean individual observation period of 3.13 years (min: 1.01 years; max: 9.94 years) within the preschool age. Overall, half of the patients received a protein restricted diet (mOTC-D: n = 3, 13.0%; fOTC-D: n = 5, 21.7%; ASS1-D: n = 4, 17.5%; ASL-D: n = 11, 47.8%), while 50% of patients received no protein restricted diet (mOTC-D: n = 6, 26.1%; fOTC-D: n = 6, 26.1%; ASS1-D: n = 7, 30.4%; ASL-D: n = 4, 17.4%). Individuals with and without a protein restricted diet did not differ with regard to their initial NH_4_^+^_max_ (*p* = 0.20; *t*-test). In addition, the degree of protein restriction was not disease-dependent in individuals receiving a protein restricted diet (*p* = 0.28; LME ANOVA), but showed an overlapping mean natural protein intake ranging from 62.95% to 77.45% WHO in mOTC-D, fOTC-D, ASS1-D, and ASL-D (Fig. 4A). In analogy, a protein restricted diet had no impact on weight gain. Neither age (*p* = 0.48; LME ANOVA) nor application of a protein restricted diet (*p* = 0.43; LME ANOVA) or interaction of both (*p* = 0.98; LME ANOVA) affected weight development (Fig. 4B). Regardless of this, symptomatic individuals receiving conservative medical management developed a progressive growth retardation (*p* = 0.008; LME ANOVA). However, this was not explained by the use of a protein restricted diet (*p* = 0.61; LME ANOVA), showing that both symptomatic individuals with or without a protein restricted diet (*p* = 0.27; LME ANOVA) experienced postnatal growth retardation (Fig. 4C). Based on 39.1% (n = 18/46) of individuals in this sample with sufficient data density, mean caloric intake does not appear to be associated with growth retardation (*p* = 0.32; LME ANOVA).

### Symptomatic UCDs have reduced to low normal plasma BCAA concentrations

Since growth retardation was independent from the use of a protein restricted diet, we wondered whether BCAA concentrations (L-valine, L-leucine, L-isoleucine) might contribute to impaired growth in symptomatic individuals with or without a protein restricted diet. Therefore, we investigated the weighted arithmetic mean values of plasma BCAAs along with the weighted arithmetic mean dosage of EAA supplements during the individual observation periods in both individuals with and without a protein restricted diet. Although individuals with a protein restricted diet received a higher weighted arithmetic mean dosage of EAA supplements (*p* = 0.03; *t*-test), concentrations of L-valine (*p* = 0.25; *t*-test), L-leucine (*p* = 0.06;* t*-test), and L-isoleucine (*p* = 0.24; *t*-test) did not differ between groups (Table 1), but were found to be reduced or at the lower end of the reference range^13^. Weighted arithmetic mean values of plasma L-arginine did not differ between both groups (*p* = 0.78; *t*-test), but were, in contrast to plasma BCAA concentrations, within the normal or upper end of the reference range^13^. Moreover, application of nitrogen scavengers (mono- or biscavenger therapy) showed a similar distribution between groups.

**Table 1:** Weighted arithmetic mean values of plasma BCAAs and L-arginine in the study sample.

	L-valine	L-leucine	L-isoleucine	L-arginine
	Mean, SD; n	Mean, SD; n	Mean, SD; n	Mean, SD; n
Reference range	133–273	64–164	31–83	38–98
**Conservative management**				
Protein restricted diet	145, 54; 23	68, 25; 23	38, 16; 23	73, 33, 23
No protein restricted diet	166, 72; 22	92, 45; 22	45, 23; 22	74, 32, 22
*p *value	0.25	0.06	0.24	0.78
**Liver transplantation**				
Prior to LTx	144, 58; 18	86, 63; 18	40, 23; 18	n/a
After LTx	224, 70; 15	123, 32; 15	67, 21; 16	n/a
*p *value	0.001	0.048	0.001	n/a

Reference ranges were defined according to (20) for children in the age range of 2–10 years and are shown in µmol/l. *P *values were calculated using a two-sided *t*-test, *p *values < 0.05 were considered significant, *n* refers to number of patients included in each group. Of note, 65% (n = 15/23) of individuals with a protein restricted diet and 59% (n = 13/22) of individuals without a protein restricted diet received (at least temporarily) supplementation with L-arginine within the observation period. BCAAs, branched-chain amino acid(s); LTx, liver transplantation; n/a, not available.

### Liver transplantation rescues postnatal growth retardation in symptomatic UCDs

**Figure 5:**
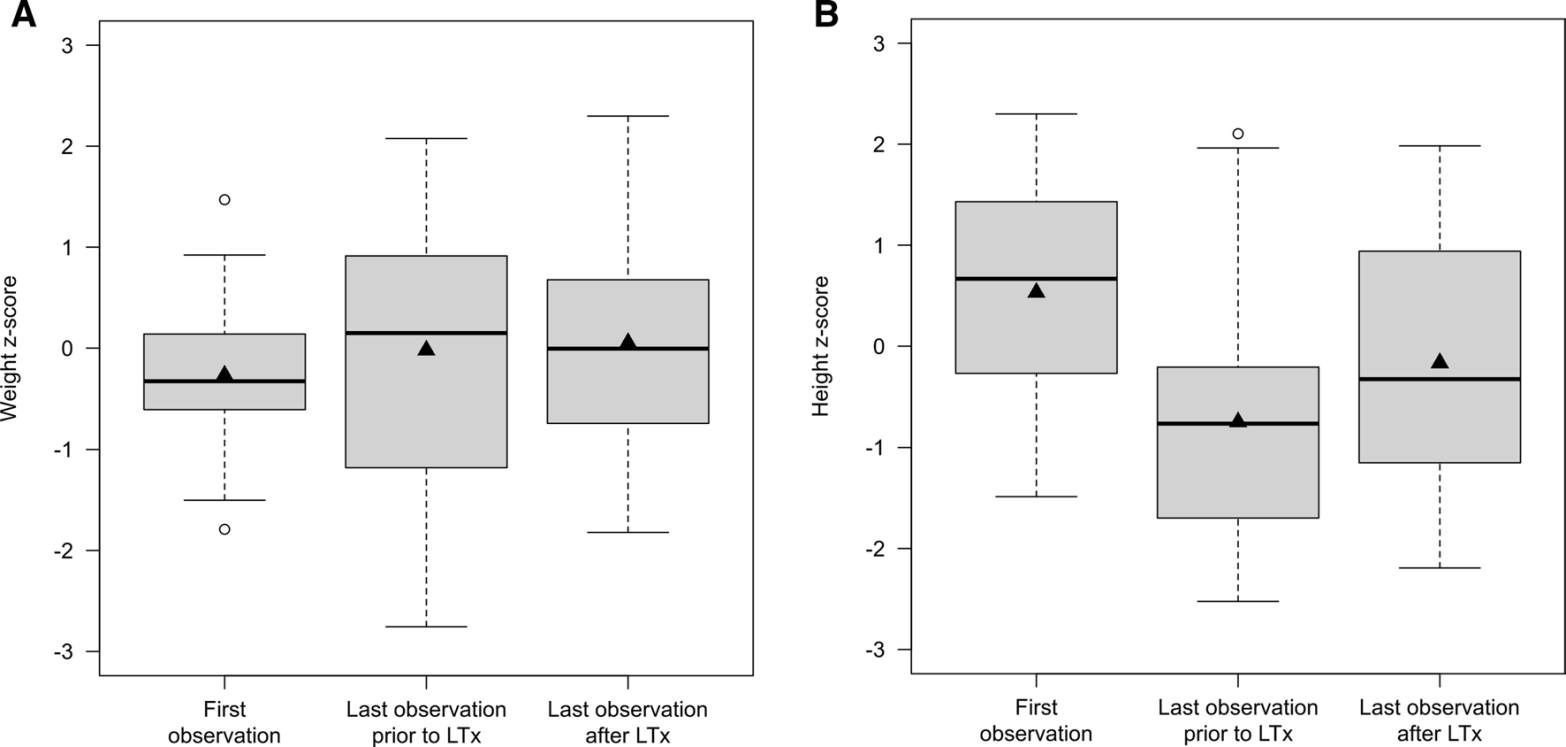
Liver transplantation appears to have a beneficial effect on growth of 19 individuals with UCDs. Individuals with UCDs (n = 19) have normal weight gain (**A**) over time pre- and post-transplantation (*p* = 0.48; LME ANOVA), but suffer from impaired growth (**B**) prior to transplantation [mean z-score at first observation: 0.53, mean z-sore at last observation prior to LTx: − 0.75; *p* < 0.001; contrast *t*-test). However, growth retardation does not further aggravate after transplantation (mean z-score at last observation prior to LTx: − 0.75, mean z-score at last observation after LTx: − 0.17; *p* = 0.13; contrast *t*-test). Data are shown as median (black line) and mean (triangle), length of the box corresponds to IQR, upper and lower whiskers correspond to max. 1.5 × IQR, each point represents an outlier. Descriptive characteristics are presented separately in Supplementary Table S1. LTx, liver transplantation.

To investigate whether LTx might exert a positive effect on growth and weight in severely affected individuals, we analyzed both endpoints of liver-transplanted individuals at three different time points, i.e. at first observation (first time point), at last observation prior to LTx (second time point), and at last observation after LTx (third time point). Overall, 19 individuals (mOTC-D: n = 7, 36.8%; ASS1-D: n = 6, 31.6%; ASL-D: n = 6, 31.6%) receiving LTx at a mean age of 2.15 years (min: 0.42 years; max: 7.76 years) were included into this analysis. Disease severity, as mirrored by initial NH_4_^+^_max_, did not differ between different diseases (*p* = 0.60; LME ANOVA). In those 19 transplanted individuals, the mean observation time of conservative management (time between first and second time point) was 1.91 years, the mean observation time after liver transplantation (time between age at transplantation and third time point) was 4.74 years. In analogy to milder affected individuals who do not undergo LTx, liver-transplanted individuals showed normal weight development (mean z-score at first observation: − 0.27, mean z-score at last observation prior to LTx: − 0.02, mean z-score at last observation after LTx: 0.05, *p* = 0.48; LME ANOVA) (Fig. 5A). In contrast, age showed an effect on linear growth (*p* < 0.001; LME ANOVA), suggesting that during conservative management patients develop a postnatal growth retardation (mean z-score at first observation: 0.53, mean z-score at last observation prior to LTx: − 0.75, *p* < 0.001; contrast *t*-test). After LTx however, linear growth of individuals stabilized along a constant z-score (mean z-score at last observation prior to LTx: − 0.75, mean z-score at last observation after LTx: − 0.17, *p* = 0.13; contrast *t*-test) (Fig. 5B). Interestingly, liver transplantation led to the elevation of all plasma BCAAs concentrations from reduced or low normal values before LTx to values well within the normal range after LTx [L-valine (*p* = 0.001; *t*-test), L-leucine (*p* = 0.048; *t*-test), L-isoleucine (*p* = 0.001; *t*-test); Table 1].

## Discussion

This study aimed at characterizing growth and weight gain in individuals with UCDs and elucidating risk factors for potentially impaired anthropometrical development. The study revealed five major results: (1) Intrauterine development in all investigated UCDs and postnatal linear growth of asymptomatic and untreated individuals were normal. (2) Symptomatic individuals were at risk of developing progressive growth impairment regardless of the underlying UCD or degree of natural protein intake. (3) Growth impairment was determined by disease severity and associated with reduced or low normal plasma BCAA concentrations. (4) LTx appeared to have a beneficial effect on growth. (5) Weight development was normal in both asymptomatic and symptomatic individuals with UCDs, regardless of the medical management.

Intrauterine growth of the investigated study sample was unaffected as indicated by anthropometrical parameters at birth which were within the normal range, thereby confirming previous data from a European cohort of individuals with UCDs^3^. In asymptomatic UCD individuals not receiving long-term management with low protein diet and nitrogen scavengers, linear growth and weight gain remained unaffected until preschool age.

In contrast, symptomatic individuals of any UCD studied receiving conservative management, particularly EO individuals, showed progressive growth impairment over time, which is in line with previous studies^14,15^, highlighting that disease severity rather than the type of UCD is a major determinant of linear growth. Since treatment intensity was shown to reflect disease severity and protein restriction has been hypothesized to be causally related to growth failure in UCDs^12^, we further investigated the impact of current treatment modalities on linear growth, particularly with regard to natural protein intake as underlying cause of potential growth retardation. Unexpectedly however, both symptomatic UCD individuals with or without protein restricted diet exhibited a progressive growth deficit, indicating that natural protein intake per se does not explain this finding. Analogously, caloric intake appeared not to affect neither weight development nor linear growth in the studied cohort. However, due to the small study sample for caloric intake, these results should be considered somewhat exploratory. Moreover, although weight development was normal in both asymptomatic and symptomatic individuals with UCDs, it remains to be elucidated whether the composition of body weight in terms of fat and lean mass might be affected by medical management.

Recently, it has been shown that supplementation of L-arginine restored plasma IGF-1 and IGF-BP3 levels in seven male individuals with LO OTC-D aged 3–5 years and gradually improved linear growth^16^. However, UCD individuals in our study cohort exhibited progressive growth failure despite plasma L-arginine levels well within the normal range independent from protein restriction or supplementation with EAAs, suggesting alternative factors causing reduced linear growth in UCDs.

Intriguingly, UCD individuals in both groups (restricted vs. non-restricted) exhibited similarly reduced or low normal plasma BCAA concentrations as indicated by the weighted arithmetic mean values for plasma L-valine, L-leucine and L-isoleucine concentrations in our study. Maintaining stable concentrations of essential and functional BCAAs is crucial to stimulate growth^17^, and deficiencies in the amino acids L-valine, L-leucine and L-isoleucine have been shown to be associated with growth failure in children^18^. Reduced BCAA concentrations are a biochemical hallmark in acute and chronic hyperammonemic conditions. Enhanced consumption of BCAAs via increased propionate oxidation as major compensatory mechanism to prevent bioenergetic impairment under acute and chronic hyperammonemic conditions has recently been elucidated^19–25^, highlighting the role of the deamination of BCAAs for the generation of succinyl-CoA (deamination of isoleucine and valine), which can supply the TCA cycle with important carbon backbones. Consistently, individuals with UCDs exhibited decreased plasma BCAA concentrations correlating with hyperammonemia^26^. Moreover, L-isoleucine and L-valine have been shown to be the only amino acids with significant cerebral uptake in patients with fulminant hepatic failure^27^, and cerebral BCAA transaminases (BCATs) are stimulated under hyperammonemic conditions, thereby enhancing the consumption of L-isoleucine and L-valine for anaplerotic reactions as well as transamination processes for the generation of L-glutamate and L-glutamine (consumption of L-valine, L-leucine and L-isoleucine) via activities of BCAT1 and BCAT2, respectively^28^. Notably, male mice hemizygous for the OTC^spf-ash^ mutation, which is characterized by chronically elevated NH_4_^+^ concentrations without spontaneously occurring acute hyperammonemic decompensations, exhibited growth failure with significantly reduced body height under regular diet when compared to their wildtype littermates at 4–6 months^29,30^, indicating that growth failure in UCD might be causatively linked to the chronically elevated NH_4_^+^ concentrations and subsequent depletion of BCAAs due to enhanced propionate oxidation. Moreover, sodium or glycerol PBA prevents the phosphorylation of the E1α subunit of the branched-chain α-keto acid dehydrogenase complex (BCKDHc) via inhibition of the BCKDHc kinase, resulting in activation of BCKDHc and increased breakdown of BCAA, resulting in synergistic reduction of plasma BCAA concentrations^7^.

In line, height z-scores in UCD patients were positively associated with patient’s plasma L-leucine (CPS1-D, mOTC-D and HHH-syndrome) and L-valine (ASS1-D and ASL-D) concentrations^14^, clearly supporting our findings. However, while the latter observation was based on a cross-sectional analysis, our results further substantiate the relevance of the identified association between BCAA depletion and growth failure by providing longitudinal data in UCDs. Of note, plasma BCAA concentrations were within the lower normal range or reduced in UCD individuals in our study, independent from the administration of EAA supplements, which has already been reported by Molema et al.^14^, indicating that the current clinical practice of prescribing EAA or BCAA supplements is not sufficiently compensating for BCAA depletion in UCD individuals. Since LTx appeared to be beneficial for the cognitive outcome in UCD individuals in a cross-sectional analysis^12,14^, we further investigated the impact of LTx on linear growth. Intriguingly, UCD individuals who exhibited growth impairment with decreasing height z-scores prior to LTx, did show a stable linear growth within a mean individual observation period of 4 years after LTx, suggesting that LTx might also prove beneficial for linear growth in UCDs. Further systematic prospective long-term follow-up investigation and increased number of analyzed individuals will be crucial to reliably determine the effect of LTx on linear growth. However, our findings are not unexpected, since LTx is an effective measure to correct the enzymatic defect thereby preventing further hyperammonemic episodes and subsequent consumption of BCAAs induced by elevated NH_4_^+^ concentrations as well as the necessity of protein restriction and long-term nitrogen scavenger therapy. Of note, this is substantiated by our finding that plasma BCAA concentrations normalized after LTx in the study sample investigated.

This analysis has several inherent limitations. While both, the E-IMD and UCDC registry contain detailed information on dietary prescriptions, they do not verify and describe the actual daily intake by a participating UCD individual or adequately control for patient compliance. Furthermore, there was no information on the quality of natural protein consumed. Practice of dietary management may vary considerably between different study centers of both consortia. Moreover, the first data entry used for different analyses might not reflect the exact time period between diagnosis and implementation of a specific treatment, which might have introduced some noise into the data due to inadequate or varying treatment modalities prior to first assessment. Next, biochemical values (plasma concentrations of NH_4_^+^, BCAAs and L-arginine) were not assessed in a central laboratory using a standardized protocol and therefore reference values differ slightly between participating study sites. There was no assessment neither of the quality of the preanalytical process nor the correctness of measurements and potential differences between contributing laboratories. Due to small sample sizes, a comparative analysis regarding the effect of a specific nitrogen scavenger (BZA vs. PBA) on plasma BCAA concentrations could not be performed and thus remains subject to future studies. Study visits in both registries usually are several months apart, therefore it has to be implied that the status recorded at any given point is representative for the preceding interval. Given the observational nature of this international, multicenter registry study, data sets can have missing values. Thus, the prospective study was performed using solely available data, which is only valid under the missing completely at random (MCAR) assumption. No methods of imputation of missing data were applied. Further intraindividual long-term follow up studies are needed, to substantiate our findings and to systematically assess linear growth in UCD individuals beyond the preschool age.

## Conclusions

Our longitudinal study reveals, that intrauterine development in all investigated UCDs as well as postnatal linear growth and weight progress of asymptomatic individuals in the absence of hyperammonemic episodes and/or conservative treatment is unaffected. In contrast, symptomatic individuals are at risk of developing progressive growth impairment over time independent from the underlying disease and the degree of natural protein restriction. Growth impairment is associated with reduced or borderline plasma BCAA concentrations. Moreover, LTx appeared to have a beneficial effect on linear growth. Future prospective clinical trials are indispensable to unequivocally prove the pathomechanistic role of BCAA depletion on impaired linear growth and potential beneficial effects of adequate EAA supplementation in UCDs.

## Materials and methods

### Eligibility criteria and overview of the UCDC and E-IMD databases

In brief, the UCDC database is registered in the U.S. National Library of Medicine (https://clinicaltrials.gov), whereas the E-IMD registry is recorded on the German Clinical Trials Register (https://www.drks.de). Requirements set forth by the ICMJE (International Committee of Medical Journal Editors) were met. All procedures were in accordance with the ethical standards of the Helsinki Declaration of 1975, as revised in 2013. The E-IMD and UCDC study protocols have received approval by the Institutional Review Board of the Medical Faculty of Heidelberg University (Ethikkommission der Medizinischen Fakultät Heidelberg, Germany; lead institution for this study, permit S-525/2010 and S-198/2011) and were also approved by all ethics committees of the participating North-American and European study sites. Written informed consent was obtained from all participants prior to inclusion in both databases. Both registries use remote data entry via electronic case report forms comprising clinical, biochemical and therapy-related data from baseline, scheduled regular follow-up investigations, and unscheduled (emergency) visits to the hospital. A detailed description of the combined and comparative research approach is provided separately^11^.

### Cornerstones and strategy for data analysis

Cut-off date for data analysis was February 25, 2019. Since the scope of this analysis comprised the effects of dietary long-term management, only data from baseline and regular follow-up visits were considered eligible. All subsequent analyses focus on individuals with OTC-D (MIM #311250), ASS1-D (MIM #215700), and ASL-D (MIM #207900) forming the study sample for this analysis, and investigating the majority of individuals with UCDs^3,11,31^. Ultra-rare UCDs with a disease-specific incidence of equal to or below 1:1,000,000^32^, such as *N*-acetylglutamate synthase deficiency, carbamolyphosphate synthetase 1 deficiency, carboanhydrase VA deficiency, arginase 1 deficiency, hyperornithinemia-hyperammonemia-homocitrullinuria syndrome, and citrin deficiency are subject to future studies. We analyzed the effect of dietary long-term management on the anthropometrical endpoints weight, height and, if available, head circumference. Since we showed recently that therapeutic intensity depends on the phenotypic severity of individuals with UCDs^12^, our study sample (individuals with mOTC-D, fOTC-D, ASS1-D, and ASL-D) was severity-adjusted. To this end, individual observation periods were defined for each patient within the study sample, reflecting a period of specific therapy intensity, and thus specific phenotypic severity. The regular visit prior to therapy escalation or therapy de-escalation corresponded to the last observation timepoint within the individuals' observation periods. Therapy escalation was defined as newly introduced application of mono- or combined scavengers, therapy de-escalation was considered as discontinuation of mono- or biscavenger therapy. Each individual within the study sample had at least two successive visits with a maximum interval of 18 months. The minimum duration of an individual observation period for each individual corresponded to 12 months. Visits within the individual observation periods provided information on biochemical values, dietary and pharmacological management, and anthropometrical endpoints.

European individuals with UCDs were compared to growth charts from the UK, since the ethnical background of the European UCD sample corresponds well to the UK^3,33^, while for North American individuals the Center for Disease Control and Prevention growth charts (https://www.cdc.gov/growthcharts/cdc_charts.htm) were used. Z-scores for height, weight, BMI and head circumference were calculated at baseline and each regular follow-up visit. Preterm infants (< 37th pregnancy week), and z-scores < -3 or > 3 were excluded from data analysis due to probably erroneous entries into the databases. Mean natural protein intake (g/kg/d), mean EAA intake (g/kg/d), and mean caloric intake (kcal/kg/d) were calculated for the patients' individual observation periods (using time weighted averages based on data from each available visit) and were indicated as percentage of WHO safe values recommendations for mean natural protein and mean EAA intake^6^, and in percentage of Food and Agriculture Organizations of the United Nations (FAO) for mean caloric intake^34^. Individuals receiving a mean natural protein intake below 100% WHO safe values during the observation period were considered to have a protein restricted diet. To monitor BCAA levels during the patients' individual observation periods, mean plasma levels for L-valine, L-leucine, L-isoleucine, and L-arginine (all in µmol/l) were calculated, where available. Moreover, use of nitrogen scavengers was documented within the individuals’ observation periods. Only individuals until the age of 18 years were included into this study.

### Inclusion and exclusion criteria

Overall, 307 individuals with UCDs were eligible for data analysis (mOTC-D: n = 82, 26.7%; fOTC-D: n = 76, 24.8%; ASS1-D: n = 78, 25.4%; ASL-D: n = 71, 23.1%). Individuals belonging to specific UCD subgroups were included in subsequent analysis, defined by distinct inclusion and exclusion criteria. Descriptive characteristics of those subgroups are presented in the corresponding results section and in Supplementary Table S1. Analysis 1 (“Anthropometrical parameters at birth are within normal range”) investigated if individuals with UCD suffered from intrauterine retardation of growth, weight, and head circumference (inclusion criteria: birth weight, length and head circumference measured within the first 2 weeks of life). Analysis 2 (“Asymptomatic individuals have a normal postnatal growth and weight development”) evaluated whether asymptomatic and untreated individuals had a normal or impaired postnatal anthropometrical development (inclusion criteria: asymptomatic individuals with UCDs; study inclusion within 12 months after age at diagnosis; no scavengers and no protein restricted diet within the individuals’ observation periods). Analysis 3 (“Symptomatic individuals have a risk of postnatal growth retardation”) investigated whether symptomatic individuals with UCDs receiving conservative management were at risk of growth and weight retardation (inclusion criteria: symptomatic individuals with UCDs, study inclusion within 12 months after age at diagnosis; exclusion criteria: asymptomatic individuals with UCDs, individuals receiving a LTx). Analysis 4 (“Postnatal growth retardation in symptomatic individuals with UCDs is not associated with a protein restricted diet”) studied whether symptomatic individuals with a protein restricted diet are at risk of developing impaired growth and weight compared to individuals without a protein restricted diet (inclusion criteria: see analysis 3, additionally only individuals with sufficient information on biochemical and therapy-related longitudinal data allowing the calculation of mean values were included; exclusion criteria: see analysis 3). Analysis 5 (“Liver transplantation rescues postnatal growth retardation in symptomatic UCDs”) investigated whether individuals receiving LTx suffered from pre- and post-transplant growth and weight retardation [inclusion criteria: individuals receiving a LTx with three distinct time points of growth and weight (first time point: first observation; second time point: last observation prior to LTx; third time point: last observation after LTx), time lag between 2nd and 3rd time point at least 12 months; exclusion criteria: individuals with conservative management].

### Data availability

The datasets generated and analyzed during the current study are not publicly available due to existing data protection laws. Furthermore, data ownership is retained by the members of the UCDC and E-IMD consortia making anonymized data available for specific research purposes. Data availability is subject to the consent of both consortia upon request.

### Statistical analysis

All statistical analyses were performed using R, a language for statistical computing and graphics (https://www.r-project.org). Multiple regression analyses were used to model a numeric dependent outcome variable with respect to several independent predictor variables. Longitudinal data was modelled with a linear mixed effect model (LME) with individuals as random factor. For multiple regression and LME, results were displayed with analysis of variance (ANOVA) tables with type two F-tests and post-hoc comparisons (contrasts) were carried out with *t*-tests based on estimated marginal means^35^. Two groups were compared with Welch two sample *t*-test, paired data with a paired *t*-test.

## Supplementary information

Supplementary information

## Abbreviations

ASx: Asymptomatic
ASL-D: Argininosuccinate lyase deficiency
ASS1-D: Argininosuccinate synthetase 1 deficiency
BCAA(s): Branched-chain amino acid(s)
BCAT(s): BCAA transaminase(s)
BMI: Body mass index
BZA: Sodium benzoate
EAA(s): Essential amino acid(s)
E-IMD: European registry and network for Intoxication type Metabolic Diseases
EO: Early onset (≤ 28 days)
fOTC-D: Female ornithine transcarbamylase deficiency
IQR: Interquartile range
LO: Late onset (> 28 days)
LTx: Liver transplantation
mOTC-D: Male ornithine transcarbamylase deficiency
NH_4_^+^_max_: Peak plasma ammonium concentration
PBA: (Sodium or glycerol) phenylbutyrate
SDS: Standard deviation score
UCDC: Urea Cycle Disorders Consortium
UCD(s): Urea cycle disorder(s)
