# Supplementary material for: Long-term effects of medical management on growth and weight in individuals with urea cycle disorders
Source: Sci Rep. 2020 Jul 20;10:11948. doi: 10.1038/s41598-020-67496-3 (PMC7371674; doi:10.1038/s41598-020-67496-3)
Supplement: Supplementary file 1 — Supplementary information [file 41598_2020_67496_MOESM1_ESM.docx]

**Long-term effects of medical management on growth and weight in individuals with urea cycle disorders**

Roland Posset^1*^, Sven F. Garbade^1*^, Florian Gleich^1^, Andrea L. Gropman^2^, Pascale de Lonlay^3^, Georg F. Hoffmann^1^, Angeles Garcia-Cazorla^4^, Sandesh C. S. Nagamani^5^, Matthias R. Baumgartner^6^, Andreas Schulze^7^, Dries Dobbelaere^8^, Marc Yudkoff^9^, Stefan Kölker^1^, Matthias Zielonka^1,10^; for the Urea Cycle Disorders Consortium (UCDC) and the European registry and network for Intoxication type Metabolic Diseases (E-IMD) consortia study group

* These authors contributed equally to the study

^1^Center for Child and Adolescent Medicine, Division of Pediatric Neurology and Metabolic Medicine, University Hospital Heidelberg, Im Neuenheimer Feld 430, 69120 Heidelberg, Germany

^2^Children's National Health System, Washington, DC, USA

^3^Hôpital Necker-Enfants Malades, Assistance Publique-Hôpitaux de Paris, Service de Maladies Metaboliques (MaMEA), filière G2M, Université Paris-Descartes, Paris, France

^4^Hospital San Joan de Deu, Institut Pediàtric de Recerca. Servicio de Neurologia and CIBERER, ISCIII, Barcelona, Spain

^5^Department of Molecular and Human Genetics, Baylor College of Medicine and Texas Children’s Hospital, Houston, Texas, USA

^6^University Children’s Hospital Zurich and Children’s Research Center, Zurich, Switzerland

^7^University of Toronto and the Hospital for Sick Children, Toronto, Ontario, Canada

^8^Centre de Référence Maladies Héréditaires du Métabolisme de l'Enfant et de l'Adulte, Jeanne de Flandre Hospital, CHRU Lille, and Faculty of Medicine, University Lille 2, Lille, France

^9^University of Pennsylvania School of Medicine and Children’s Hospital of Philadelphia, Philadelphia, Pennsylvania, USA

^10^Heidelberg Research Center for Molecular Medicine (HRCMM), Heidelberg, Germany

**🖂 Corresponding author:**

Matthias Zielonka, MD

Center for Pediatric and Adolescent Medicine

Division of Pediatric Neurology and Metabolic Medicine

University Hospital Heidelberg

Im Neuenheimer Feld 430, 69120 Heidelberg, Germany

Phone: +49 6221 56 4002

Fax: +49 6221 56 6391

E-mail: [Matthias.Zielonka@med.uni-heidelberg.de](mailto:Matthias.Zielonka@med.uni-heidelberg.de)

**Supplementary Information**

**Supplementary Table S1. Descriptive characteristics of the study cohort per analysis.**

| **Anthropometrical parameters at birth are within normal range (Figure 1)** | | | | | | |
| --- | --- | --- | --- | --- | --- | --- |
|  | **Overall** | **mOTC-D** | **fOTC-D** | | **ASS1-D** | **ASL-D** |
| **Weight (z-score)**  Mean, SD  Median [Q1, Q3]  Min, Max; n | -0.24, 0.83  **-0.22** [-0.81, 0.30]  -2.30, 2.00; 205 | -0.30, 0.73  **-0.35** [-0.88, 0.13]  -1.68, 1.50; 54 | -0.41, 0.91  **-0.34** [-1.13, 0.24]  -2.24, 1.45; 39 | | -0.07, 0.73  **-0.06** [-0.55, 0.62]  -2.30, 1.12; 64 | -0.28, 0.97  **-0.36** [-1.00, 0.17]  -1.79, 2.00; 48 |
| **Length (z-score)**  Mean, SD  Median [Q1, Q3]  Min, Max; n | 0.14, 1.01  **0.00** [-0.50, 0.67]  -2.94, 2.61; 205 | 0.09, 0.87  **0.26** [-0.65, 0.52]  -1.74, 2.17; 54 | -0.08, 0.96  **0.00** [-0.52, 0.55]  -2.51, 1.50; 39 | | 0.32, 0.96  **0.27** [-0.30, 0.80]  -1.96, 2.61; 64 | 0.13, 1.21  **0.00** [-0.50, 0.69]  -2.94, 2.61; 48 |
| **Head circumference  (z-score)**  Mean, SD  Median [Q1, Q3]  Min, Max; n | -0.13, 1.04  **0.00** [-0.78, 0.47]  -2.64, 2.73; 205 | -0.39, 0.83  **-0.37** [-0.77, 0.08]  -2.24, 1.50; 54 | 0.08, 1.28  **0.00** [-0.94, 0.88]  -2.64, 2.64; 39 | | 0.00, 0.97  **0.00** [-0.75, 0.75]  -2.24, 1.76; 64 | -0.18, 1.11  **-0.40** [-0.88, 0.27]  -2.44, 2.73; 48 |
| **Asymptomatic individuals have a normal postnatal growth and weight development (Figure 2)** | | | | | | |
|  | **Overall** | **mOTC-D** | **fOTC-D** | | **ASS1-D** | **ASL-D** |
| **Individual observation period (years)**  Mean, SD  Median [Q1, Q3]  Min, Max; n | 2.96, 1.21  **2.62** [1.96, 4.05]  1.59, 4.54; 11 | 1.78, 0.27  **1.78** [1.69, 1.88]  1.59, 1.98; 2 | 2.86, 1.45  **2.10** [2.02, 3.32]  1.94, 4.54; 3 | | 2.82, 0.91  **3.05** [2.37, 3.50]  1.61, 3.56; 4 | 4.54, 0.00  **4.54** [4.54, 4.54]  4.54, 4.54; 2 |
| **Weight (z-score)**  **First observation**  Mean, SD  Median [Q1, Q3]  Min, Max; n | 0.09, 1.23  **0.44** [-0.77, 0.66]  -1.77, 2.55; 11 | -0.67, 1.56  **-0.67** [-1.22, -0.11]  -1.77, 0.44; 2 | -0.40, 1.11  **-0.59** [-0.99, 0.11]  -1.40, 0.80; 3 | | 0.77, 1.45  **0.74** [0.16, 1.36]  -0.96, 2.55; 4 | 0.21, 0.33  **0.21** [0.10, 0.33]  -0.02, 0.44; 2 |
| **Weight (z-score)**  **Last observation**  Mean, SD  Median [Q1, Q3]  Min, Max; n | 0.65, 1.11  **0.11** [-0.03, 1.70]  -0.93, 2.28; 11 | 0.27, 0.36  **0.27** [0.14, 0.40]  0.01, 0.53; 2 | 0.56, 1.39  **-0.08** [-0.23, 1.04]  -0.38, 2.16; 3 | | 1.19, 1.45  **1.70** [0.87, 2.01]  -0.93, 2.28; 4 | 0.10, 0.02  **0.10** [0.09, 0.11]  0.08, 0.11; 2 |
| **Height (z-score)**  **First observation**  Mean, SD  Median [Q1, Q3]  Min, Max; n | 0.02, 0.91  **-0.10** [-0.67, 0.71]  -1.45, 1.28; 11 | -0.77, 0.96  **-0.77** [-1.11, -0.44]  -1.45, -0.10; 2 | -0.36, 0.63  **-0.51** [-0.71, -0.09]  -0.91, 0.33; 3 | | 0.60, 0.99  **0.98** [0.33, 1.26]  -0.83, 1.28; 4 | 0.23, 0.67  **0.23** [-0.01, 0.47]  -0.24, 0.71; 2 |
| **Height (z-score)**  **Last observation**  Mean, SD  Median [Q1, Q3]  Min, Max; n | 0.62, 0.91  **0.54** [0.00, 0.98]  -0.60, 2.26; 11 | 0.64, 0.15  **0.64** [0.59, 0.70]  0.54, 0.75; 2 | 0.75, 1.31  **0.12** [0.00, 1.19]  -0.13, 2.26; 3 | | 0.54, 1.26  **0.38** [-0.48, 1.40]  -0.60, 1.97; 4 | 0.55, 0.08  **0.55** [0.52, 0.58]  0.50, 0.61; 2 |
| **Symptomatic individuals have a risk of postnatal growth retardation (Figure 3)** | | | | | | |
|  | **Overall** | **mOTC-D** | **fOTC-D** | | **ASS1-D** | **ASL-D** |
| **Individual observation period (years)**  Mean, SD  Median [Q1, Q3]  Min, Max; n | 4.81, 3.21  **4.29** [1.90, 6.77]  1.00, 12.44; 130 | 4.32, 2.97  **3.31** [1.91, 5.89]  1.01, 12.00; 33 | 4.96, 3.15  **4.77** [2.35, 6.44]  1.03, 11.35; 42 | | 5.20, 3.55  **4.10** [2.21, 6.58]  1.24, 12.44; 25 | 4.81, 3.35  **4.43** [1.70, 7.26]  1.00, 12.04; 30 |
| **Initial NH_4_^+^_max_ (µmol/l)**  Mean, SD  Median [Q1, Q3]  Min, Max; n | 525, 625  **290** [190, 572]  27, 3600; 130 | 436, 606  **239** [168, 420]  27, 3369; 33 | 276, 174  **229** [157, 327]  39, 959; 42 | | 747, 691  **631** [226, 1091]  41, 2900; 25 | 787, 825  **385** [260, 1024]  142, 3600; 30 |
|  | **EO** | | | | **LO** | |
| **Initial NH_4_^+^_max_ (µmol/l)**  Mean, SD  Median [Q1, Q3]  Min, Max; n | 864, 819  **652** [266, 1146]  95, 3600; 55 | | | | 276, 208  **230** [154, 329]  27, 1561; 75 | |
| **Weight development: β-coefficient** | -0.01 | | | | 0.02 | |
| **Height development: β-coefficient** | -0.11 | | | | -0.02 | |
| **BMI development: β-coefficient** | 0.11 | | | | 0.03 | |
| **Postnatal growth retardation in symptomatic individuals with UCDs is not associated with a protein restricted diet (Figure 4)** | | | | | | |
|  | **Overall** | **mOTC-D** | **fOTC-D** | | **ASS1-D** | **ASL-D** |
| **Individual observation period (years)**  Mean, SD  Median [Q1, Q3]  Min, Max; n | 3.13, 2.20  **2.27** [1.45, 4.22]  1.01, 9.94; 46 | 2.27, 1.42  **1.91** [1.34, 2.50]  1.01, 5.50; 9 | 3.80, 2.97  **2.38** [1.47, 5.24]  1.15, 9.94; 11 | | 3.42, 1.92  **3.47** [1.90, 4.18]  1.24, 7.06; 11 | 2.93, 2.13  **2.14** [1.45, 3.49]  1.02, 8.01; 15 |
|  | **No protein restricted diet** | | | **Protein restricted diet** | | |
| **Initial NH_4_^+^_max_ (µmol/l)**  Mean, SD  Median [Q1, Q3]  Min, Max; n | 500, 604  **275** [209, 366]  95, 2373; 18 | | | 807, 860  **332** [249, 1033]  142, 3369; 21 | | |
| **Natural protein intake (% WHO)**  Mean, SD  Median [Q1, Q3]  Min, Max; n | 145.1, 67.4  **126.9** [108.0, 145.6]  103.1, 353.7; 23 | | | 72.2, 17.1  **73.6** [61.2, 84.7]  42.1, 99.8; 23 | | |
| **Weight development: β-coefficient** | -0.01 | | | -0.01 | | |
| **Height development: β-coefficient** | -0.06 | | | -0.03 | | |

|  | **Overall** | **mOTC-D** | **fOTC-D** | **ASS1-D** | | **ASL-D** |
| --- | --- | --- | --- | --- | --- | --- |
| **Natural protein intake for protein restricted diet (% WHO)**  Mean, SD  Median [Q1, Q3]  Min, Max; n | 72.2, 17.1  **73.6** [61.2, 84.7]  42.1, 99.8; 23 | 66.6, 29.7  **56.9** [49.9, 78.4]  42.9, 99.8; 3 | 71.3, 10.6  **73.6** [61.8, 74.1]  60.6, 86.4; 5 | 63.0, 14.1  **65.7** [54.8, 73.9]  45.2, 75.1; 4 | | 77.5, 17.0  **78.3** [67.7, 89.7]  42.1, 99.2; 11 |
| **Liver transplantation rescues postnatal growth retardation in symptomatic UCDs (Figure 5)** | | | | | | |
|  | **Overall** | **mOTC-D** | **ASS1-D** | | **ASL-D** | |
| **Age at first observation (years)**  Mean, SD  Median [Q1, Q3]  Min, Max; n | 0.00, 0.00  **0.00** [0.00, 0.00]  0.00, 0.00; 19 | 0.00, 0.00  **0.00** [0.00, 0.00]  0.00, 0.00; 7 | 0.00, 0.00  **0.00** [0.00, 0.00]  0.00, 0.00; 6 | | 0.00, 0.00  **0.00** [0.00, 0.00]  0.00, 0.00; 6 | |
| **Age at last observation prior to LTx (years)**  Mean, SD  Median [Q1, Q3]  Min, Max; n | 1.91, 1.97  **1.22** [0.54, 2.29]  0.21, 7.56; 19 | 1.00, 1.39  **0.33** [0.27, 0.94]  0.21, 4.04; 7 | 2.20, 2.69  **1.22** [0.79, 1.93]  0.41, 7.56; 6 | | 2.67, 1.59  **2.29** [1.66, 3.26]  1.01, 5.39; 6 | |
| **Age at LTx (years)**  Mean, SD  Median [Q1, Q3]  Min, Max; n | 2.15, 1.95  **1.34** [0.92, 2.55]  0.42, 7.76; 19 | 1.30, 1.43  **0.78** [0.49, 1.23]  0.42, 4.44; 7 | 2.50, 2.67  **1.55** [1.08, 2.37]  0.60, 7.76; 6 | | 2.80, 1.55  **2.40** [1.82, 3.44]  1.15, 5.42; 6 | |
| **Age at last observation after LTx (years)**  Mean, SD  Median [Q1, Q3]  Min, Max; n | 6.89, 2.25  **6.59** [5.40, 8.31]  1.39, 11.00; 19 | 6.35, 3.03  **6.12** [5.16, 7.83]  1.39, 11.00; 7 | 7.15, 2.13  **7.05** [5.34, 8.97]  4.96, 9.49; 6 | | 7.24, 1.44  **7.16** [6.48, 7.74]  5.36, 9.54; 6 | |
| **Initial NH_4_^+^_max_ (µmol/l)**  Mean, SD  Median [Q1, Q3]  Min, Max; n | 996, 697  **1003** [351, 1614]  131, 2279; 19 | 1209, 671  **1387** [836, 1636]  131, 2000; 7 | 926, 632  **773** [418, 1511]  291, 1662; 6 | | 817, 838  **527** [191, 1136]  150, 2279; 6 | |

| **Weight (z-score)**  **First observation**  Mean, SD  Median [Q1, Q3]  Min, Max; n | -0.27, 0.86  **-0.33** [-0.60, 0.14]  -1.79, 1.47; 19 | -0.26, 0.92  **0.04** [-0.97, 0.32]  -1.50, 0.92; 7 | -0.10, 0.46  **-0.21** [-0.38, 0.09]  -0.62, 0.68; 6 | -0.44, 1.17  **-0.45** [-1.24, -0.05]  -1.79, 1.47; 6 |
| --- | --- | --- | --- | --- |
| **Weight (z-score)**  **Last observation prior to LTx**  Mean, SD  Median [Q1, Q3]  Min, Max; n | -0.22, 1.41  **0.15** [-1.18, 0.91]  -2.76, 2.08; 19 | -0.59, 1.68  **-1.10** [-1.66, 0.54]  -2.76, 1.95; 7 | 0.59, 1.22  **0.64** [0.14, 1.36]  -1.39, 2.08; 6 | 0.04, 1.15  **0.22** [-0.74, 0.88]  -1.58, 1.34; 6 |
| **Weight (z-score)**  **Last observation after LTx**  Mean, SD  Median [Q1, Q3]  Min, Max; n | 0.05, 1.15  **-0.01** [-0.74, 0.68]  -1.82, 2.30; 19 | -0.12, 1.44  **-0.33** [-1.20, 0.71]  -1.82, 2.30; 7 | 0.22, 1.09  **0.21** [0.00, 0.63]  -1.57, 1.75; 6 | 0.09, 1.01  **-0.15** [-0.32, 0.30]  -1.11, 1.87; 6 |
| **Height (z-score)**  **First observation**  Mean, SD  Median [Q1, Q3]  Min, Max; n | 0.53, 1.13  **0.67** [-0.27, 1.43]  -1.49, 2.30; 19 | 0.51, 0.92  **0.54** [0.07, 1.22]  -1.09, 1.53; 7 | 1.00, 1.34  **1.39** [0.77, 1.72]  -1.49, 2.30; 6 | 0.09, 1.15  **-0.24** [-0.83, 0.93]  -0.98, 1.72; 6 |
| **Height (z-score)**  **Last observation prior to LTx**  Mean, SD  Median [Q1, Q3]  Min, Max; n | -0.75, 1.28  **-0.76** [-1.70, -0.21]  -2.52, 2.10; 19 | -1.52, 0.82  **-1.66** [-2.13, -0.98]  -2.52, -0.26; 7 | 0.58, 1.20  **0.33** [-0.38, 1.58]  -0.65, 2.10; 6 | -1.18, 0.65  **-1.28** [-1.69, -0.83]  -1.83, -0.16; 6 |
| **Height (z-score)**  **Last observation after LTx**  Mean, SD  Median [Q1, Q3]  Min, Max; n | -0.17, 1.25  **-0.33** [-1.15, 0.94]  -2.19, 1.98; 19 | -0.49, 1.03  **-0.56** [-1.13, 0.04]  -1.84, 1.16; 7 | 0.90, 0.80  **1.07** [0.53, 1.23]  -0.35, 1.98; 6 | -0.86, 1.28  **-1.15** [-1.81, 0.04]  -2.19, 0.95; 6 |

Descriptive characteristics for results section with subheadings shaded in grey. Statistical analyses are presented in the body of the manuscript. Z-scores were calculated using the normative data for the European (13) or North American sample (<https://www.cdc.gov/growthcharts/cdc_charts.htm>). ASL-D argininosuccinate lyase deficiency; ASS1-D, argininosuccinate synthetase 1 deficiency; EO, early onset; fOTC-D, female ornithine transcarbamylase deficiency; LO, late onset; LTx, liver transplantation; mOTC-D, male ornithine transcarbamylase deficiency; n/a, not available; NH_4_^+^_max_, peak plasma ammonium concentration.

**Supplementary Table S2. Individual contributors from the UCDC and E-IMD consortia study group**

| **UCDC consortium (to be listed in PubMed in alphabetical order)** |
| --- |
| Nicholas Ah Mew, Children's National Health System, Washington, DC, USA |
| Susan A. Berry, University of Minnesota, Minneapolis, Minnesota, USA |
| Shawn E. McCandless, Children’s Hospital Colorado and University of Colorado School of Medicine, Aurora, Colorado, USA |
| Curtis Coughlin, Children’s Hospital Colorado and University of Colorado School of Medicine, Aurora, Colorado, USA |
| Gregory Enns, Stanford Children’s Health, 730 Welch Road, Palo Alto, CA 94304 |
| Renata C. Gallagher, University of California, San Francisco, 550 16th Street, San Francisco, CA 94143 |
| Lindsay Burrage, Department of Molecular and Human Genetics, Baylor College of Medicine and Texas Children’s Hospital, Houston, Texas, USA |
| Jennifer Seminara, Children’s National Health System, Washington, DC, USA |
| Cary O. Harding, Oregon Health and Science University, Portland, Oregon, USA |
| Peter Burgard, Center for Pediatric and Adolescent Medicine, Division of Pediatric Neurology and Metabolic Medicine, University Hospital Heidelberg, Im Neuenheimer Feld 430, 69120 Heidelberg, Germany |
| Cynthia Le Mons, National Urea Cycle Disorders Foundation, USA |
| J. Lawrence Merritt II, University of Washington and Seattle Children’s Hospital, Seattle, Washington, USA |
| Tamar Stricker, University Children’s Hospital Zurich, Zurich, Switzerland |
| Jirair K. Bedoyan, Center for Human Genetics and Department of Genetics and Genome Sciences, University Hospitals Cleveland Medical Center and Case Western Reserve University, Cleveland, Ohio, USA |
| Gerard T. Berry, Harvard Medical School and Boston Children’s Hospital, Boston, Massachusetts, USA |
| George A. Diaz, Mount Sinai School of Medicine, Department of Genetics and Genomics Sciences, New York, NY, USA |
| Derek Wong, David Geffen School of Medicine at UCLA, Los Angeles, California, USA |
| Mendel Tuchman, Children’s National Health System and The George Washington School of Medicine, Washington, District of Columbia, USA |
| Susan Waisbren, Harvard Medical School and Boston Children’s Hospital, Boston, Massachusetts, USA |
| James D. Weisfeld-Adams, Section of Clinical Genetics and Metabolism, Department of Pediatrics, University of Colorado School of Medicine, Aurora, CO, USA |

| **E-IMD registry (to be listed in PubMed in alphabetical order)** |
| --- |
| Alberto B. Burlina, Division of Inherited Metabolic Diseases, Reference Centre Expanded Newborn Screening, Department of Woman's and Child's Health, University Hospital Padova, Italy |
| Elisa Leão Teles, Unidade de Doenças Metabólicas, Serviço de Pediatria, Hospital de S. João, EPE, Porto, Portugal |
| Consuelo Pedrón-Giner, Division of Gastroenterology and Nutrition, Hospital Infantil Universitario Niño Jesús, Madrid, Spain |
| Allan M. Lund, Centre Inherited Metabolic Diseases, Departments of Paediatrics and Clinical Genetics, Copenhagen University Hospital, Rigshospitalet, Copenhagen, Denmark |
| Carlo Dionisi-Vici, Ospedale Pediatrico Bambino Gesù, U.O.C. Patologia Metabolica, Rome, Italy |
| Monique Williams, Erasmus MC-Sophia Kinderziekenhuis, Erasmus Universiteit Rotterdam, Rotterdam, The Netherlands |
| Ulrike Mütze, Center for Pediatric and Adolescent Medicine, Division of Pediatric Neurology and Metabolic Medicine, University Hospital Heidelberg, Im Neuenheimer Feld 430, 69120 Heidelberg, Germany |
| Daniela Karall, Clinic for Pediatrics, Division of Inherited Metabolic Disorders, Medical University of Innsbruck, Innsbruck, Austria |
| Javier Blasco-Alonso, Hospital Materno-Infantil, AVda Arroyo de los Ángeles s/n; 29011 , Málaga, Spain |
| Maria L. Couce, Hospital Clinico Universitario de Santiago de Compostela, Metabolic Unit, Department of Pediatrics, Santiago de Compostela, Spain |
| Jolanta Sykut-Cegielska, Institute of Mother and Child, Department of Inborn Errors of Metabolism and Paediatrics, Warsaw, Poland |
| Persephone Augoustides-Savvopoulou, 1st Pediatric Department, Aristotle University of Thessaloniki, Thessaloniki, Greece |
| Angeles Ruiz Gomez, Metabolic Diseases Unit, Pediatric Neurology Department, Hospital Universitario Son Dureta, Palma de Mallorca, Spain |
| Ivo Barić, University Hospital Center Zagreb and University of Zagreb, School of Medicine, Zagreb, Croatia |
| Manuel Schiff, Robert-Debré University Hospital, Reference Center for Inborn Errors of Metabolism, Paris, France |
| Yin-Hsiu Chien, Department of Medical Genetics and Pediatrics, National Taiwan University Hospital, National Taiwan University College of Medicine, Taipei, Taiwan. |
| Martin Lindner, University Children’s Hospital Frankfurt, Frankfurt, Germany |
| Brigitte Chabrol, Centre de Référence des Maladies Héréditaires du Métabolisme, Service de Neurologie, Hôpital d’Enfants, CHU Timone, Marseille, France |
| Anastasia Skouma, Institute of Child Health, Athens, Greece |
| Jiri Zeman, Department of Paediatrics, First Faculty of Medicine and General Faculty Hospital, Prague, Czech Republic |
| Etienne Sokal, Cliniques Universitaires St Luc, Université Catholique de Louvain, Service Gastroentérologie and Hépatologie Pédiatrique, Bruxelles, Belgium |
| René Santer, University Medical Center Eppendorf, Hamburg, Germany |
| Francois Eyskens, Universitair Ziekenhuis Antwerpen, Antwerpen, Belgium |
| Peter Freisinger, Klinik für Kinder- und Jugendmedizin, Klinikum am Steinenberg, Reutlingen, Germany |
| Luis Peña-Quintana, Gastroenterology and Nutrition Unit Complejo Hospitalario Universitario Insular-Materno Infantil, CIBEROBN, Las Palmas de Gran Canaria University, Las Palmas, Spain |
| Dominique Roland, Institut de Pathologie et de Génétique ASBL, Centre Agréé des Maladies Héréditaires du Métabolisme, Centre de Génétique Humaine, Avenue Georges Lemaitre, 25, 6041, Gosselies, Belgium |
| Elisenda Cortès-Saladelafont, Department of Paediatrics Universitat Autònoma de Barcelona, Badalona, Spain |
| Maja Djordjevic, Institut za zdravstvenu zastitu majke i deteta Srbije “Dr Vukan Cupic”, Radoja Dakica Street 6-8, Novi Beograd, and University of Belgrade, School of Medicine, Republic of Serbia |
